# Exploring Barriers and Facilitators to Self‐Management for Patients With Persistent Musculoskeletal Conditions Following NHS‐Led Hydrotherapy: A Service Evaluation

**DOI:** 10.1002/msc.70075

**Published:** 2025-02-26

**Authors:** Natasha Pathak, Roger Newham, Neil Smith, Roanna Burgess

**Affiliations:** ^1^ Musculoskeletal Physiotherapy Department Sandwell and West Birmingham NHS Trust West Bromwich UK; ^2^ School of Nursing Institute of Clinical Sciences University of Birmingham Birmingham UK

**Keywords:** barriers, facilitators, hydrotherapy, musculoskeletal, persistent, self‐management, service evaluation

## Abstract

**Introduction:**

Persistent musculoskeletal (MSK) disorders are one of the leading reasons for years lived with disability within the UK. Guidelines encourage integrating self‐management support. Hydrotherapy supports patients with persistent MSK conditions; however, self‐management following NHS‐led hydrotherapy has been inconsistent.

**Aim:**

To identify outcomes of hydrotherapy alongside the barriers, facilitators, and contributory factors affecting self‐management in the local persistent MSK pain population.

**Methods:**

Between April 2023 and May 2023, a service evaluation was undertaken exploring factors affecting self‐management in the local persistent MSK pain population following NHS‐led hydrotherapy. Demographics, clinical factors, functional status (MSK‐HQ) and patient‐reported experiences, including barriers and facilitators to self‐management, were recorded. Data was analysed using descriptive statistics alongside exploration of themes.

**Results:**

Ninety patients completed hydrotherapy. White British (*n* = 24) and Indian British (*n* = 23) were the most common groups to attend. Multiple joint (*n* = 27) and spinal conditions (*n* = 26) were the most common MSK conditions. Mean pre‐ and post‐MSK‐HQ scores were 20.8 and 26.1, respectively. Among the contactable patients (*n* = 69), 49 patients did not maintain independent water‐based self‐management reporting barriers such as ongoing support, access and financial concerns. Patients supported the establishment of a support group (*n* = 56) to help maintain water‐based exercises.

**Conclusions:**

The local persistent MSK pain population lacks self‐efficacy for independent water‐based self‐management. A supportive and collaborative approach is proposed to address this via a patient‐led hydrotherapy support group.

## 1 Background

### 1.1 Musculoskeletal Disorders Burden

In 2019, the ‘Global Burden of Disease’ study identified musculoskeletal (MSK) disorders as being among the top ten drivers for increased disability resulting in an escalating demand on the healthcare system (Institute for Health Metrics and Evaluation (IHME), 2020). An estimated one‐third of the United Kingdom (UK) population report persistent and disabling MSK pain (IHME 2020). There is growing concern that primary care consultations have been heavily utilised by persistent MSK pain patients with approximately 20% of the UK population seeking care for this issue (Hill et al. 2022). In the presence of persistent and disabling MSK conditions, there is an elevated risk of adopting unhealthy behaviours, experiencing poorer disability outcomes and having an overall decline in quality of life (The King's Fund, 2018).

MSK disorders have recently been recognised as a major health condition in the ‘Major Conditions Strategy (MCS)’ (Department of Health and Social Care (DHSC), 2023). MSK disorders can include conditions such as fibromyalgia, inflammatory pathologies, osteoarthritis and osteoporosis. The MCS (DHSC 2023) and NHS England (2023a) encourage supporting patients with long‐term MSK conditions through a targeted and individualised approach tailored to their needs and preferences. This involves providing seamless and integrated self‐management pathways to improve population health, which is actively endorsed in persistent pain management guidelines (National Institute for Clinical Excellence (NICE), 2021). Evidence shows that patient empowerment and personalised care improve long‐term management, patient satisfaction and quality of life in individuals with persistent pain (Barratt, 2018; NHS England, 2019; The King's Fund, 2018).

### 1.2 Personalised Care: The Role of Social Determinants

Personalised care is deemed crucial to the health deterioration prevention agenda (Chartered Society of Physiotherapy (CSP), 2021); however, socioeconomic factors can become barriers to personalised care, resulting in poorer health and disability outcomes (The King's Fund, 2018). Studies show that social deprivation increases the likelihood of long‐term MSK conditions (CSP 2023). The Marmot review (Marmot et al. 2020) highlights the importance of preventative measures in maintaining health and addressing socioeconomic disparities, resulting in sustainably healthier communities. There is a growing concern that populations with heightened levels of socioeconomic deprivation and disability experience worse outcomes (Arthritis and Musculoskeletal Alliance (ARMA), 2024) and lower levels of adherence to self‐management strategies (Van Hecke et al. 2017).

### 1.3 The Local Population

The demographics discussed within this service evaluation is the Sandwell borough in the West Midlands. According to Sandwell Trends (2019), Sandwell is ranked as the 12th most deprived borough in the UK with 48% of residents identifying from ethnic minority groups. The West Midlands exhibits the highest levels of inactivity in the UK, with approximately 28.6% of its population being inactive (Sport England, 2020). ARMA (2021) and NHS England (2023a) promote focussing on populations experiencing significant socioeconomic deprivation to alleviate the strain on the NHS and MSK services. To do this, we need to understand the barriers that this population faces to enable a personalised self‐management approach that is both seamless and well‐integrated.

### 1.4 Self‐Management Barriers and Support Requirements: Literature Exploration

Self‐management plans (SMPs) are endorsed in the guidelines (NICE 2021); however, there is a paucity of research exploring this topic in persistent MSK conditions. A recent systematic review did not report significant beneficial outcomes in persistent MSK conditions (Elbers et al. 2018).

A recent systematic review explored the barriers and facilitators of self‐management in the persistent MSK pain population from the patients' perspective and it was concluded that one of the main themes was the lack of accessible and supportive self‐management strategies (Spink, Wagner, and Orrock 2021). Knowledge, understanding and physical and psychological factors were also themes that influenced self‐management in this population (Spink, Wagner, and Orrock 2021). Alternatively, Farley (2020) conducted an integrative review identifying health literacy, access and support as the main barriers to developing self‐efficacy skills enabling self‐management. The suggested strategies to promote self‐efficacy were centred around mobile apps, social media platforms and self‐management programmes.

According to research, approximately 50%–70% of patients with persistent pain are non‐adherent to their exercise management strategy beyond formal intervention (Peek et al. 2016). Written exercise information, goal planning and targeted behavioural programmes were enablers for self‐management (Peek et al. 2016). Other studies have investigated the influence of health inequality on the self‐management of long‐term conditions and found that individuals from deprived areas exhibited decreased adherence to self‐management initiatives (Van Hecke et al. 2017; Hardman, Begg and Spelton 2020).

### 1.5 NHS‐Led Hydrotherapy: Service Evaluation Rationale

Sandwell and West Birmingham NHS Trust's (SWB) hydrotherapy service, based in the Sandwell borough, manages patients with persistent MSK pain.

Literature shows that hydrotherapy can improve pain, quality of life and function in the short‐term in the persistent MSK pain population (Andrade et al. 2019; NICE 2021); however, the long‐term benefits of hydrotherapy and continuity beyond NHS‐led care have not been evaluated.

The hydrotherapy service provides teaching sessions aiming to facilitate independent self‐management following formal NHS‐led hydrotherapy sessions. Previous service evaluations have highlighted that despite positive outcomes, patients with persistent MSK pain conditions often do not maintain independent self‐management. This highlights that this patient population does not adopt the necessary self‐efficacy skills to enable self‐management of their long‐term conditions; however, the reasons for this are unclear.

### 1.6 Aims

This evaluation aimed to identify the outcomes of hydrotherapy alongside barriers, facilitators, contributory factors and patient characteristics affecting self‐management adherence in the local persistent MSK pain population following a course of hydrotherapy. These data will allow stakeholders to devise a plan for quality improvement (QI) that is tailored to this demographics.

## 2 Methods

### 2.1 Study Design

This service evaluation was registered with the SWB NHS Trust (ID: 2424, Date: 16/07/2023) and approved by the Trust's ‘Research and Development Team’ and the ‘Clinical Effectiveness Team.’

The Standards for Quality Improvement Reporting Excellence 2.0 (SQUIRE 2.0) guidelines were used in reporting this service evaluation. The Plan‐Do‐Study‐Act (PDSA) cycle was used as a framework to implement changes aimed at enhancing healthcare quality (NHS England, 2022).

The ‘Plan’ stage consisted of a proposal to the service leads. The ‘Do’ stage involved capturing data from patients who had completed a course of hydrotherapy. Six weeks after the completion of hydrotherapy sessions, patients were contacted by telephone to gather feedback on their ability to maintain independent water‐based exercises. Data was analysed in the ‘Study’ phase of the cycle. Finally, in the ‘Act’ stage, conclusions and clinical implications were drawn to plan an iterative process analysing need for change.

### 2.2 Setting and Participants

This evaluation was conducted in an NHS Trust in the West Midlands, UK. The ‘Community MSK Service’ manages the care of the adult population with MSK conditions. The hydrotherapy department is based at a local leisure centre. Patients with persistent MSK pain received four hydrotherapy sessions. This evaluation included patients aged 18 and above who were referred and successfully completed all four hydrotherapy sessions.

### 2.3 Data Collection

Quantitative and qualitative data, including Patient Reported Outcome Measures (PROMs) and Patient Reported Experience Measures (PREMs), were routinely collected from patients with persistent MSK pain who were referred to the hydrotherapy service in April and May 2023. The patients who completed their hydrotherapy sessions and completed PROMs and PREMs were included in this evaluation. The inclusion of this dataset was based on convenience, aiming to provide a manageable set for analysis. Table 1 summarises this data.

**TABLE 1:** Summary of data collected.

Data type	Response option	Capture point/Source
Cohort evaluation		
Age	Continuous numeric	Baseline/Electronic patient record
Gender	Categorical (4 options) (Male; female; other; undisclosed)	Baseline/Electronic patient record
Ethnicity	Categorical (16 options) (British mixed; White British; other White; White Irish; White Irish/Black Caribbean; White Asian; White Black African; Indian British; pakistani British; Bangladeshi British; Caribbean; Black British; African; Chinese; other; not specified)	Baseline/Electronic patient record
Deprivation score	Categorical (10 options) 1 (most deprived) and 10 (least deprived)	Baseline/Electronic patient record IMD calculated using postcode. Via http://www.fscbiodiversity.uk/imd
Clinical factors		
Pain Site	Categorical (9 options) (Knee; ankle; hip; spine; shoulder; neck; hand/wrist; elbow; multiple joint)	Baseline/Electronic patient record
Previous hydrotherapy	Binary (Yes; No)	Baseline/Electronic patient record
Documented self‐management plan (SMP)	Binary (Yes; No)	Baseline/Electronic patient record
Functional status		
MSK‐HQ	Categorical (15 questions) (Scored out of 56)	Baseline (pre MSK‐HQ) and endpoint (post MSK‐HQ)/Questionnaire
PREMs, barriers & facilitators		
Benefit of hydrotherapy	Categorical (10 options) 1 (least beneficial) to 10 (most beneficial)	Endpoint/Questionnaire
Continued independent water‐based exercises after 6 weeks?	Categorical (4 options) (Yes completely; Yes, to some extent; no; unable to contact/declined consent)	Six weeks post endpoint/Telephone
If not, then what would have helped you to sustain independent water‐based exercise?	Free text/conversation	Six weeks post endpoint/Telephone
Do you feel that a patient‐led support group would help you to sustain independent water‐based exercise?	Categorical (4 options) (Yes definitely; Yes, to some extent; No not at all; Don't know/not sure)	Six weeks post endpoint/Telephone

Burgess et al. (2021) suggest gathering a set of core metrics for utilisation in the UK community and primary care MSK services. The Musculoskeletal Health Questionnaire (MSK‐HQ) is a validated tool that assesses MSK health status with a high score (0–56) indicating superior MSK health (Hill et al. 2016). ‘The Index of Multiple Deprivation 2015’ (Department for Communities and Local Government, 2015) measures relative deprivation in England ranking areas in deciles from 1 (most deprived) to 10 (least deprived) considering various domains including education, employment, health, disability, crime, income and living conditions.

Patient cohort evaluation, clinical factors, PROMs and PREMs were recorded on a Microsoft Excel spreadsheet for individuals referred to the hydrotherapy service. At the initial hydrotherapy appointment, patients completed MSK‐HQ scores (pre MSK‐HQ). On the final hydrotherapy appointment, MSK‐HQ scores were reassessed (post MSK‐HQ) and the individuals expressed their perceived benefit from the service. Six weeks post‐session completion, consenting patients were contacted by one author via telephone to explore PREMs (see Table 1). Trust interpreters were used where language barriers existed. Verbatim responses were documented. ‘Yes, to some extent’ was noted for patients who started independent water‐based self‐management but not regularly, whereas ‘Yes completely’ was noted for patients who completed regular self‐management. Three attempts were made to contact patients for PREM feedback; thereafter, 'unable to contact/declined consent’ was noted on the spreadsheet.

### 2.4 Data Analysis

Individuals who did not complete their hydrotherapy sessions were excluded from this evaluation. PREM responses were recorded verbatim, and topics/themes were formed. Descriptive statistics were used to describe cohort data, clinical factors, PROMs and PREMs.

## 3 Results

### 3.1 Project Steps Changes

Although patients were questioned regarding self‐management enablers, their responses were more focussed on self‐management barriers. These responses were recorded verbatim forming barrier topics. Further probing questions were not used. These barrier topics were not qualitatively analysed given the scope of this evaluation. Emerging topics prompted the inclusion of a question regarding patients' views on a patient‐led support group and the probability of maintaining independent water‐based self‐management through this intervention. This aimed to explore the factors enabling self‐management within this group and promoting personalised care.

Ninety patients successfully completed hydrotherapy sessions. Data for 21 patients was unavailable as they either nationally opted out of being contacted for evaluation purposes (*n* = 12) or remained non‐contactable for PREM feedback following three attempted telephone calls (*n* = 9). These patients were not included in the PREM evaluation.

### 3.2 Cohort Evaluation

**FIGURE 1:**
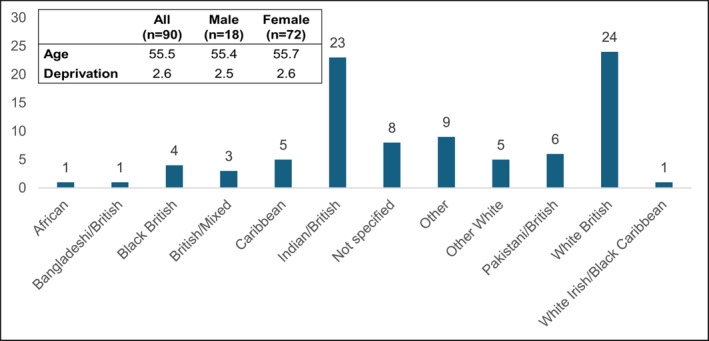
Cohort evaluation data (*n* = 90).

Figure 1 displays cohort data and deprivation scores.

### 3.3 Clinical Factors

**FIGURE 2:**
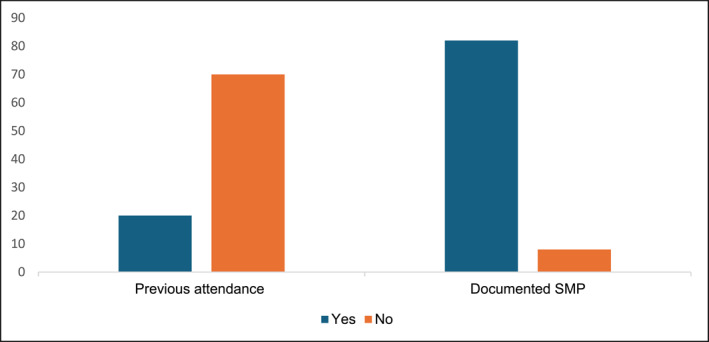
Previous attendance and documented SMP (*n* = 90).

Figure 2 shows the number of patients who had previously attended hydrotherapy sessions and the number of patients with a documented self‐management plan (SMP).

### 3.4 Functional Status

Table 2 details MSK‐HQ health status scores according to the presenting MSK pain condition. The difference between these scores is labelled ‘MSK‐HQ change.’ Patients with persistent hip pain had the lowest pre MSK‐HQ score (18.7), whereas those with persistent shoulder pain had the highest pre MSK‐HQ score (24.0). The smallest mean MSK‐HQ change was observed for persistent shoulder pain patients (3.1), whereas the highest was noted for persistent ankle pain patients (10.0).

**TABLE 2:** Overview of MSK‐HQ scores (*n* = 90).

	Ankle (*n* = 4)	Elbow (*n* = 1)	Hip (*n* = 6)	Knee (*n* = 18)	Multiple joint (*n* = 27)	Shoulder (*n* = 8)	Spine (*n* = 26)	Overall (*n* = 90)
Mean pre MSK HQ	22.5	21.0	18.7	21.9	19.9	24.0	20.3	20.8 ± 8.2
Mean post MSK HQ	32.5	30.0	28.3	28.1	23.4	27.1	25.4	26.1 ± 9.5
Mean MSK HQ change	10.0	9.0	9.7	6.1	3.6	3.1	5.0	5.2 ±5.3
Perceived benefit (/10)	9.8	8.0	7.7	7.6	7.6	9.0	7.2	7.7

### 3.5 Patient Reported Experience Measures

Table 2 illustrates the perceived benefit of hydrotherapy intervention based on the patients' MSK pain conditions. The most significant benefit was among patients with persistent ankle and shoulder pain scoring 9.8 and 9.0, respectively. Persistent spinal pain patients experienced the least benefit (7.2).

### 3.6 Barriers and Facilitators

**FIGURE 3:**
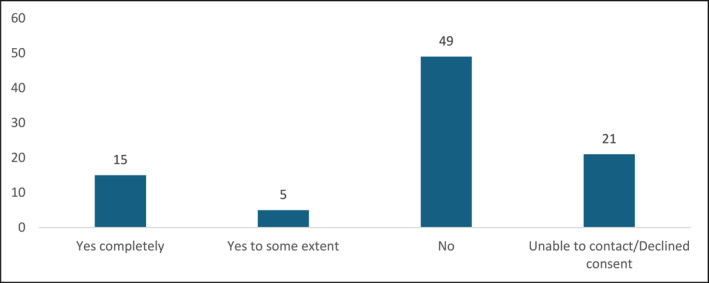
Patients' self‐management approach (*n* = 90).

Figure 3 depicts the number of patients who either maintained or did not maintain independent water‐based self‐management following hydrotherapy sessions. Table 3 illustrates self‐management maintenance among patients categorised by ethnicity.

**TABLE 3:** Self‐management approach categorised by ethnicity (*n* = 69).

	No	Yes (to some extent)	Yes (completely)	Total
African	0	0	1	1
Bangladeshi/British	1	0	0	1
Black/British	1	0	0	1
British/Mixed	1	0	0	1
Caribbean	4	0	0	4
Indian/British	11	3	2	16
Not Specified	4	0	1	5
Other	5	2	1	8
Other White	3	0	4	5
Pakistani/British	1	0	4	5
White British	17	0	5	22
White Irish/Caribbean	1	0	0	1
Total	49	5	15	69

Table 4 presents examples of qualitative data collected from 69 contactable patients demonstrating their connection to emerging barrier topics.

**TABLE 4:** Qualitative data linking to barrier topics (*n* = 69).

Barrier topics	Patient response examples
Ongoing support (*n* = 15)	“Four sessions in the water was not enough for me.” “The sessions were not enough for my problem but helped.”
Cost (*n* = 15)	“I am on benefits so I do not have the money for it but I enjoyed it.” “If it was cheaper then I would have tried to carry on.”
Access (*n* = 11)	“I can't get to my local pool as it is closed.” “I was getting better but my pool is closed.”
No change (*n* = 6)	“It made no difference to me.” “I didn't find it helpful for me.”
Pain (*n* = 5)	“My pain stops me from being more active.” “I can't cope with the pain.”
Other (*n* = 1)	“My wife has been ill so I have not had the time to go.”
Too easy (*n* = 1)	“It was easy for me.”

**FIGURE 4:**
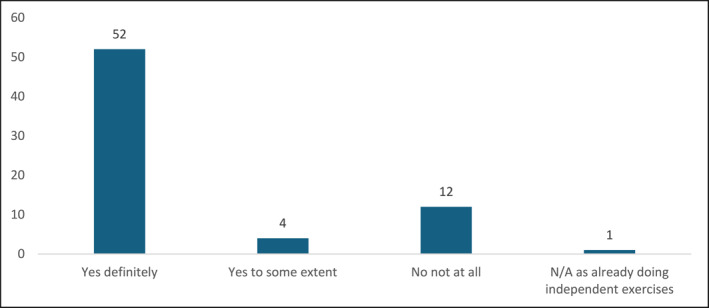
Patients' perceptions on the necessity of a patient‐led support group (*n* = 69).

All 69 contactable patients were asked an additional question regarding their perception of whether a patient‐led support group would enable maintenance of independent water‐based self‐management. Figure 4 displays these results.

Approximately 81% (*n* = 56) of contactable patients expressed that a patient‐led support group would either definitely or to some extent assist them in maintaining independent water‐based self‐management beyond NHS‐led sessions.

## 4 Discussion

### 4.1 Summary

This service evaluation is the first of its kind that evaluates service outcomes alongside the barriers, facilitators, contributory factors and patient characteristics affecting independent water‐based self‐management adherence in the local persistent MSK pain population.

### 4.2 Cohort Evaluation

Predominantly female patients with an average age of 55.5 attended this hydrotherapy self‐management intervention. This is akin to a recent systematic review of self‐management interventions in MSK pain (Healey et al. 2023) where 70% of attendees were females with an average age of 58.5. Similarly, other research echoes that 19.3% of females suffer with MSK conditions compared to 14.6% of males, and 38% of women suffer with chronic pain, whilst only 30% of men report chronic pain in England (Office for Health Improvement and Disparities (OHID), 2022). Therefore, the sample of patients included within this evaluation is similar to the wider persistent pain population.

Of the 82 patients with a defined ethnic group, 24 patients identified as White British with the remaining 58 identifying from a BAME community. Nationally, 14% of the population is estimated to be from a BAME demographic; however, this figure is much higher than the national average in Sandwell and West Birmingham where BAME communities make up 40% of the population (NHS Black Country Integrated Care Board (ICB), 2024). These statistics align with the results from this service evaluation, as largely BAME communities accessed hydrotherapy. In England, patients from Pakistani, Black Caribbean and White British communities are more likely to report persistent MSK conditions at 20.8%, 18.7% and 16.8%, respectively (OHID 2022). This was not evident from this dataset, with most patients identifying as White British or Indian British. This highlights system and people barriers in accessing hydrotherapy self‐management intervention in groups more likely to report persistent MSK conditions. This could be due to cultural sensitivity to attending hydrotherapy sessions, as some patients might not consider hydrotherapy as a self‐management strategy due to the need for swimwear.

Average deprivation scores of 2.6 were observed within this dataset. Individuals from low socioeconomic backgrounds suffer health inequalities and a higher prevalence of long‐term MSK conditions (OHID 2022). Low health literacy contributes to health inequalities. Health literacy is crucial in making informed decisions regarding wellbeing (Guo et al. 2023). Systematic reviews have found that low health literacy is responsible for poor self‐management behaviours and increasing healthcare demands, thereby affecting the uptake, access, and effectiveness of self‐management interventions (Mackey et al. 2016; Healey et al. 2023). The results from this evaluation align with existing literature, indicating low self‐management uptake among patients primarily from BAME and low socioeconomic backgrounds. A feasible argument for this could be that patients with low health literacy may struggle to understand their complex condition and thereby lack the empowerment skills to engage in managing their MSK condition long‐term. This needs to be considered when planning discharge and self‐management support packages for patients within Sandwell and West Birmingham.

### 4.3 Clinical Factors

According to OHID (2022), the most prevalent MSK conditions in England are multiple joint pain (55%), back pain (42%) and neck and shoulder pain (24%). This is reflected within this evaluation, where spinal and multiple joint pains were among the most common conditions. The ‘MSK Service Standards’ recommend developing an SMP for patients with long‐term MSK conditions (CSP 2021). SMPs enable a shared decision‐making process by developing self‐efficacy skills tailored to specific preferences. It is interesting to note that most patients had a documented SMP, which included hydrotherapy as a self‐management strategy; however, uptake of independent water‐based self‐management remained low. Research shows that discussing an SMP with patients does not affect self‐efficacy in persistent condition management; however, it did affect self‐monitoring skills (Eikelenboom et al. 2016). From this evaluation, it can be inferred that possessing a documented and mutually agreed SMP does not guarantee self‐management engagement or development of self‐efficacy skills. Additionally, 20% of patients had previously attended hydrotherapy for their persistent MSK conditions, further highlighting poor self‐efficacy skills in this demographics.

### 4.4 Functional Status

Average pre MSK‐HQ scores of 20.8 in the local population were much lower than those in other published musculoskeletal studies. A national first contact practice evaluation found that average MSK‐HQ scores were 33.8 (Stynes et al. 2021); however, these results are limited to participants identifying from a White ethnic background, which is not representative of the local population. In contrast, Hill et al. (2016) found that patients from five West Midlands towns in the UK presented with an average MSK‐HQ score of 28.62; however, this study explored scores in heterogenous MSK conditions, lacking ethnically diverse representation and in areas with less deprivation than in Sandwell and the Black Country ICB. The Black Country is the second most deprived ICB nationally (NHS Black Country ICB 2024). In summary, the local population's MSK health on average is worse than in other reported studies indicating high MSK complexity; however, there is paucity in the literature that explores MSK‐HQ scores from BAME and low socioeconomic populations.

According to the research, it can be argued that patients from this evaluation showed small functional changes in MSK‐HQ scores, which could explain poor self‐management uptake. Given that these patients presented with persistent MSK pain conditions, achieving complete pain resolution is unlikely following four formal NHS‐led hydrotherapy sessions; however, hydrotherapy may only have been a part of a patient's healthcare episode due to the multifactorial complexity of persistent MSK pain conditions. An umbrella review of outcome predictors in MSK care found that lower baseline health status scores were strong predictors of poor functional outcomes (Burgess et al. 2020), aligning with the results from this evaluation. Furthermore, according to research, the average MSK‐HQ difference of 5.2 in this cohort did not meet the expected minimal clinically important difference (MCID) of 5.5 (Price et al. 2019; Scott et al. 2020). Other studies have found that clinically meaningful improvement on discharge predicted self‐efficacy for self‐management (Souza et al. 2020). This again could explain poor self‐management uptake in this population; however, MSK‐HQ MCID has not been formally researched in under‐represented groups. Given the low baseline scores in this evaluation, an average MSK‐HQ change score of 5.2 could be significant in this population if outcomes were case‐mix adjusted for complexity alongside other hydrotherapy services (Burgess, Lewis, and Hill 2023). This highlights the need for enhanced health intelligence in this setting to allow for benchmarking of service outcomes to support targeted QI.

Interestingly, one patient had an MSK‐HQ change score of minus ten, indicating worsening MSK health following hydrotherapy; however, this patient also reported eight out of ten as their perceived benefit of hydrotherapy. Another patient reported an MSK‐HQ change score as high as 26; however, this participant still did not continue with independent water‐based self‐management stating cost as a barrier. Furthermore, some patients' MSK‐HQ scores were as low as three and four, with some selecting the option in the MSK‐HQ questionnaire indicating being unable to walk, wash and dress themselves independently; nonetheless, the same patients walked to the leisure centre for their sessions and were able to wash and dress after the sessions without assistance. Additionally, the results show that shoulder patients make the smallest change in MSK‐HQ scores but report significant benefit from the intervention. A feasible argument for these reported scores could be poor language proficiency as a barrier to completing PROMs correctly (Ocloo et al. 2021). Literature shows that PROMs do not always translate easily into other languages without taking cultural context into account (Prinsen et al. 2018). Currently, there is no evidence to suggest that the MSK‐HQ can be translated into common languages observed in this region, such as Punjabi, Urdu, or Polish. In conclusion, this evaluation highlights that self‐efficacy for self‐management goes beyond functional scores in the local population.

### 4.5 Barriers and Facilitators

This population was willing to disclose self‐management barriers rather than facilitators highlighting their attitude to pain and illness. A recent systematic review supports this evaluation, indicating that ongoing support from practitioners or peers can facilitate self‐management with accessibility, affordability, and availability being key drivers for self‐management (Spink, Wagner, and Orrock 2021). Within this review, it was identified that psychosocial variables can reduce self‐management motivation.

The data from this evaluation displays a clear requirement for the development of a peer‐support group aimed at sustaining ongoing water‐based self‐management. NHS England (2023b) support establishment of peer support within self‐management strategies by developing knowledge, skills, and emotional resilience. NHS England (2023b) recommend embedding peer‐supported self‐management in pathways making them accessible, safe, inclusive, adaptive, flexible, and personalised. A recent qualitative study explored the impact of peer support groups through semi‐structured interviews (Farr et al. 2021). They found that social and mutual understanding not only provided effective self‐management strategies but also helped in averting setbacks, which can be common in persistent pain. Furthermore, a recent service evaluation found that the implementation of a community‐based aquatic support group was not only a feasible option but also met with positive acceptance from patients with MSK disorders (Wilson et al. 2024). These peer‐supported groups are low cost, can reduce the NHS burden, and provide long‐term social recovery, which is essential for the management of long‐term MSK conditions.

### 4.6 Limitations

Several colleagues collected and entered data into the spreadsheet and a second reviewer was not utilised to verify the accuracy of the entered data. Following the development of barrier topics, there were no additional probing questions regarding self‐management enablement, as qualitative analysis was limited by the scope of this service evaluation.

The self‐reporting nature of the MSK‐HQ could have introduced a systematic bias. Although interpreters were utilised, language barriers, language proficiency and lack of direct translation may have affected the outcomes. Furthermore, this population had much lower MSK‐HQ scores than average; therefore, direct comparison in the available literature is challenging.

Access to missing PREM data from patients who nationally opted out of being contacted or were non‐contactable may have impacted the results affecting their validity and reliability. Additionally, a larger data sample could have yielded more comprehensive results. Finally, given the scope of service evaluations and small numbers across conditions and groups, these results are not generalisable, as it is a local evaluation exploring specific barriers faced by the local complex, deprived and diverse population.

### 4.7 Implications

Setting up a patient‐led support group will aim to support the service in driving forward this population's personalised care agenda, building upon self‐efficacy skills in this population and reducing the local MSK challenges and healthcare costs. The QI initiative will require shared innovation going beyond traditional boundaries, prompting collaboration with patients, social enterprises, voluntary sectors, stakeholders, and community leisure centres, enabling population health improvement. Furthermore, this evaluation has highlighted that MSK‐HQ scores vary in low socioeconomic and BAME groups; therefore, there are onward implications for further formal research to include more diverse populations to support QI in real‐world settings.

## 5 Conclusion

This evaluation has achieved the intended objectives of understanding the outcomes of hydrotherapy and the local persistent MSK pain population's approach to maintenance of self‐management following NHS‐led hydrotherapy. Plausible factors influencing poor self‐management uptake were explored, such as low health literacy, poor language proficiency, inadequate self‐efficacy skills and below average functional scores.

In addition, this evaluation identified the local population's barriers to independent self‐management such as ongoing support, financial constraints, access issues, pain and lack of functional change. The local population's preference appears to lean towards collaborative and supported self‐management approaches to empower them to continue with water‐based exercise.

## Author Contributions

**Natasha Pathak:** conceptualisation (lead), data curation (lead), formal analysis (lead), methodology (lead), writing–original draft preparation (lead), writing–review and editing (lead). **Roger Newham:** supervision (lead), validation (supporting), writing–review and editing (supporting), formal analysis (supporting). **Neil Smith:** conceptualisation (supporting), formal analysis (supporting), methodology (supporting), validation (supporting), writing–review and editing (supporting), supervision (supporting). **Roanna Burgess:** conceptualisation (supporting), methodology (supporting), validation (supporting), writing–review and editing (supporting).

## Ethics Statement

This project was deemed to be a service evaluation; therefore, ethical approval was not required (See Appendix I). In accordance with the routine local service protocol, only patients agreeing to participate in service feedback were contacted.

## Conflicts of Interest

The authors declare no conflicts of interest.

## Data Availability

The data that support the findings of this study are available on request from the corresponding author. The data are not publicly available due to privacy or ethical restrictions.
